# Extrapolative microRNA precursor based SSR mining from tea EST database in respect to agronomic traits

**DOI:** 10.1186/s13104-017-2577-x

**Published:** 2017-07-06

**Authors:** Anjan Hazra, Nirjhar Dasgupta, Chandan Sengupta, Sauren Das

**Affiliations:** 1 Agricultural and Ecological Research Unit, Indian Statistical Institute, 203, Barrackpore Trunk Road, Kolkata, 700 108 India; 2 Department of Botany, University of Kalyani, Nadia, Kalyani, 741235 India

**Keywords:** Micro RNA, Simple sequence repeats, Tea quality, Trait specific marker

## Abstract

Tea (*Camellia sinensis*, (L.) Kuntze) is considered as most popular drink across the world and it is widely consumed beverage for its several health-benefit characteristics. These positive traits primarily rely on its regulatory networks of different metabolic pathways. Development of microsatellite markers from the conserved genomic regions are being worthwhile for reviewing the genetic diversity of closely related species or self-pollinated species. Although several SSR markers have been reported, in tea, the trait-specific Simple Sequence Repeat (SSR) markers, leading to be useful in marker assisted breeding technique, are yet to be identified. Micro RNAs are short, non-coding RNA molecules, involved in post transcriptional mode of gene regulation and thus effects on related phenotype. Present study deals with identification of the microsatellite motifs within the reported and predicted miRNA precursors that are effectively followed by designing of primers from SSR flanking regions in order to PCR validation. In addition to the earlier reports, two new miRNAs are predicting here from tea expressed tag sequence database. Furthermore, 18 SSR motifs are found to be in 13 of all 33 predicted miRNAs. Trinucleotide motifs are most abundant among all followed by dinucleotides. Since, miRNA based SSR markers are evidenced to have significant role on genetic fingerprinting study, these outcomes would pave the way in developing novel markers for tagging tea specific agronomic traits as well as substantiating non-conventional breeding program.

## Introduction

Tea (*Camellia sinensis*) is most popularly consumed beverage across the world and being a cash-crop, it receives much attention to the scientific community. The vibrant research interest basically stands for its massive demand to health conscious people for its antioxidant and a broad spectrum therapeutic potentiality [1, 2]. China is the largest tea producer as well as exporter preceding by India and Sri Lanka [3]. The fermented tea or black tea is the most common among all different types of tea consumed, although antioxidant and other health benefit properties lies maximum on non-fermented green tea [4]. As a result, demand for quality tea has increased much to the end users and tea planters. Challenges for exploring superior cultivars with better agronomic traits are still point of interest to the researchers.

Molecular marker assisted technique in breeding programme for the selection or development of cultivars with desired trait from a large population is well established [5]. Among different markers used in crop improvement and molecular breeding technique, microsatellite markers are profoundly used for its reliability and time saving method. Moreover, due to being co-dominant, abundant, hyper-variable and co-operative to high-throughput analysis, microsatellite markers are considered as ideal for plant genetic linkage mapping, physical mapping, population studies, genotype identification and crop improvement [6]. Predominant of such markers like SSR, ISSR, EST-SSR have been effectively utilized in several crop improvement program [7–11]. MicroRNA precursor based SSR markers are very recently incorporated in this chapter and mostly utilized in marker trait association analysis in several species [12–15]. These micro RNAs are short, non-coding RNA molecules, involved in post transcriptional mode of gene regulation and [16] thus effects on related phenotype [17, 18]. There are a large number of molecular markers available for tea so far [19], however a very few are reported to be linked with some specific trait [20]. Therefore exploration and characterization of novel and already available markers are of prime point of interest. Considering the above, present study aims to identify the microsatellite motifs within the reported and predicted miRNA precursors that are effectively followed by designing of primers for PCR validation.

## Materials and methods

### Retrieval of data, filtering and trimming

Already predicted tea miRNA candidates were fished out from available literature [21–23]. Furthermore, to screen if any pre miRNA were within the tea EST database updated so far (November 2016), standard methodology of miRNA screening [24] were followed with some minor customization. All available reported miRNAs of viridiplantae from online repository miRBase v21.0 [25] and the entire EST collection of tea were retrieved from NCBI dbEST [26], followed by elimination of redundant sequences and trimming polyA tails using PRINSEQ v0.20.4 [27].

### Prediction of miRNAs

The set of published miRNAs were used for a homology search against tea EST collection and the best hits with a minimum length of 18 nucleotides and a maximum miRNA length cover up to 26 nucleotides and not more than 3 mismatches were taken for further analysis. After elimination of protein coding transcripts utilizing BLASTx [28], the remaining candidates were subjected to the prediction of stem-loop structure using Mfold [29] to check possibility of their pre-miRNA existence. The potential miRNA was mined considering the criteria: (a) position of mature miRNA on arm of the hairpin, (b) minimum paired residues in miRNA = 14 and unpaired residues not more than = 5, (c) maximum number of G–U pairs in miRNA = 5, (d) maximum bulge size of 3nt, (e) the negative minimal folding free energy (MFE) is low (≤−18 kcal/mol) [22], and (f) minimal folding free energy index (MFEI = [(MFE/length of the RNA sequence) * 100]/(G+C)%) is high (>0.85) [30–32].

### MicroRNA target predictions and their function

The exclusively predicted miRNAs were analyzed for their putative target genes employing the psRNATarget server [33] with default parameters. Subsequently, to recognize the functions of such predicted targets, they were undergone BLAST programme in NCBI. Finally a complete list was prepared taking all previously and presently reported tea miRNAs with their putative function.

### Exploration of SSRs within predicted microRNAs

The simple sequence repeat motifs within all available and reported pre-miRNA sequences were investigated by the Websat online program [34]. The parameters were set for identifying perfect di-, tri-, tetra-, penta-, and hexa-nucleotide motifs with minimum repeat numbers of 6, 4, 3, 3 and 3 respectively.

### Designing primers from SSR flanking region

The primer pairs from SSR flanking regions were designed with BatchPrimer3 server [35]. For the same, parameters were set as follows: length range = 18–23 nucleotides with 21 as optimum; PCR product size range = 100–400 bp [36]; optimum annealing temperature = 55 °C; and GC content 40–60%, with 50% as optimum.

## Result and discussion

**Fig. 1:**
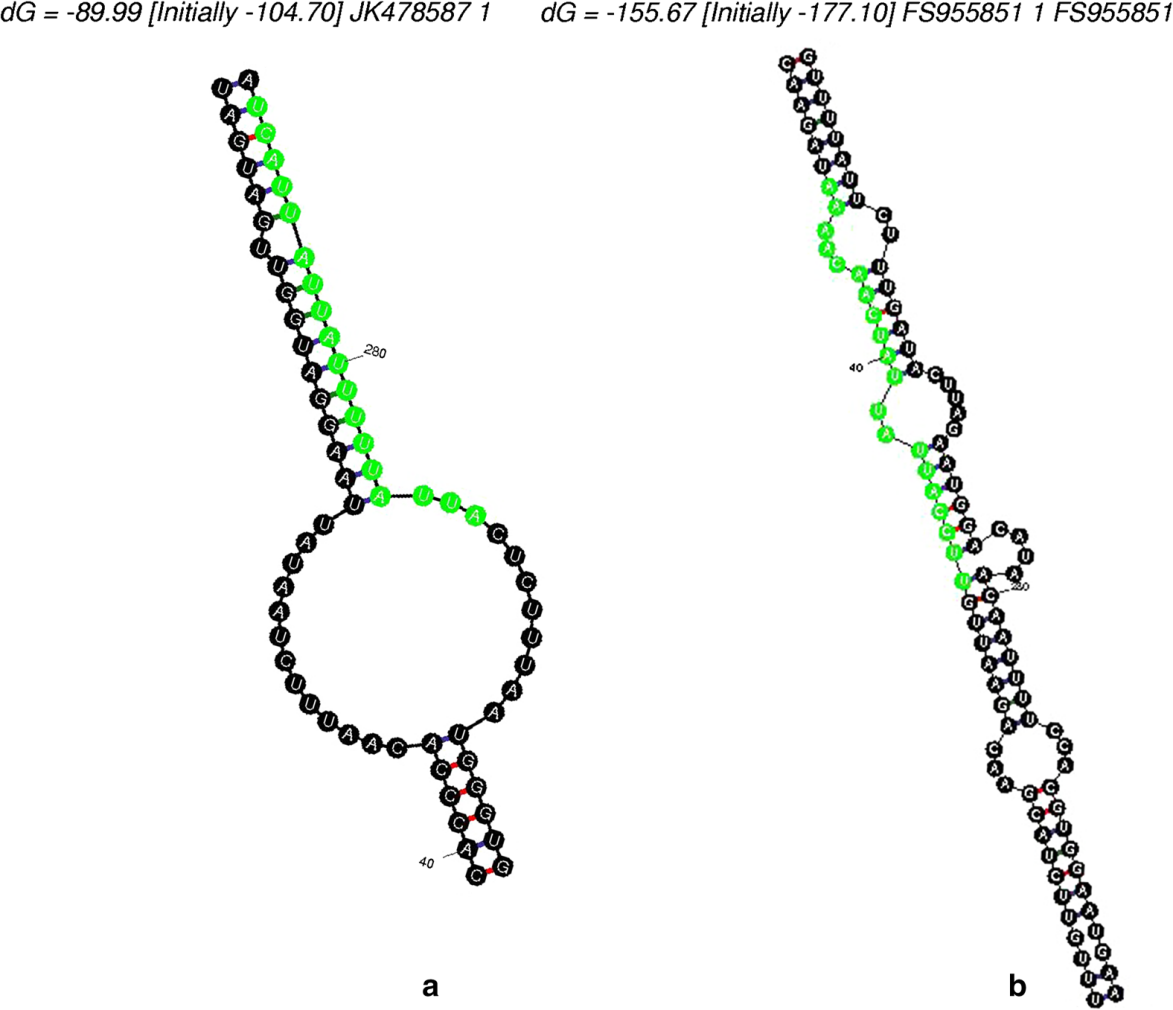
The predicted secondary step-loop structures of new tea pre-miRNAs with mature miRNA sequence highlighted in *green*

In the present study, previously reported miRNA sequences were utilized to find their homolog ones from tea as it is already known that plant mature miRNAs are highly conserved within the plant kingdom, and miRNA genes in one species may exist as orthologs or homologs in other species [30, 37]. With the help of this hypothesis, known miRNAs were utilized to discover novel potential miRNAs in tea. All 8442 miRNAs reported from viridiplantae so far were utilized and after elimination of 3676 exact duplicates using bioinformatics tool, a non-redundant collection was taken for further analysis. Similarly, tea EST collection of 49,670 sequences were made into a non-redundant 40,686 numbers. The BLAST search could fish out 52 number of ESTs with required level of homology i.e. minimum 18nt length similarity with not more than 3 mismatches. A total 15 of them already reported earlier as pre-miRNA [21–23]. Leftover candidates were analyzed through Mfold program as the miRNA precursors should be able to form stem-loop hairpin in their secondary structure for processing by Dicer enzyme [38] and subsequently possible false miRNA precursors were manually removed. In present study two more potential pre-miRNA (Fig. 1) were identified with accession JK478587.1 and FS955851.1 for having miR1533 and miR8002-3p respectively.

The identified pre-miRNAs belonging to two families had sequence lengths of 419 and 787 bp respectively (Table 1). Length variation was also evident in previous report [22, 39, 40]. Beside, commonly studied parameter in miRNA prediction is the minimum free energy (MFE) level which indicates the stability of RNA secondary structure [32] and longer pre-miRNA sequences generally have lower MFEs for maintaining its stability [41]. Here MFE values calculated by Mfold server were −89.99 and −155.67 kcal (Table 1). The MFE index or MEFI values were also calculated to distinguish miRNA from other RNAs precisely [41, 42]. Accordingly, plant pre-miRNA should have a MFEI greater than 0.85, whereas mRNAs, tRNAs, and rRNAs have a lower MFEI. In this study both identified candidate had MEFI values more than 1 indicating there possible existence in reality. Moreover sequence alignment of the new tea based miRNAs with its homolog ones from reported data showed only the initial 1 nucleotide was missing in case of miR1533 and a few nucleotides were missing from both ends and a single mismatch in entire length of miR8002-3p that strengthens the findings of this computational prediction.

**Table 1:** List of predicted microRNAs and occurrence of SSR motifs with designed primer

Predicted miRNA	Precusor id	MFE (−kCal)	References	SSR start	SSR end	SSR motif	SSR length	Primer F	Primer R	Product size
miR164	CV013669	20	[21]							
miR169	GE650220	7.8	[21]	81	93	CGC	3	GGAGTGAAGGAGGGAAGA	CCGAGAGAATAATAAGGAGGA	205
miR1846	DN976181	40.5	[21]							
miR1863	GH623864	19.2	[21]	224	236	GAG	3	AGTGATTATTGGTGGTGGTC	TCTCAACCAATTCAACAAGTC	152
miR 408	GD254786.1	120.1	[22]	519	541	TA	2	CTGTTACTGCAGCTTAACCAA	AAATATGCTGCTCATTCAAAC	152
				142	154	GGC	3	AGAAGATGTCTCAGGGAAGAG	ACACAAGTATGTCACCAGCTC	177
csi-miR1171	FE943069.1	45.97	[22]	280	295	GTGGA	5	GAACCTTTCCTCCAGAATTTA	TCACATTTAGCTTTTCACTCC	162
csi-miR414a	GD254734.1	63.18	[22]							
csi-miR414d	GW342817.1	37.72	[22]	170	188	GATGAC	6	ACGATGGTGGTTATGAATATG	GGGTTTTTGTTAAGTTGTTCA	139
csi-miR414f	GE651542.1	18.5	[22]	99	111	TTC	3	AGACAAAAACCAAGGCTAGAT	CATCTTGTGCAGATCTCAGTT	149
				121	139	ATC	3			
cas-miR1122	EU849076.1	91.23	[22]							
csi-miR414 g	CV013826.1	25.2	[22]	504	519	CTC	3	TGATGATGAGGAAGGAGATAA	TTGCTTTAGTGAAACAACTCC	136
csi-miRf10132-akr	CV014169.1	69.5	[22]							
cja-miR2910	U42815.1	−91	[22]							
csi-miR2914	AB120309.1	20.9	[22]							
cas-miRf10185-akr	GH623933.1	51.1	[22]							
cas-miR11590-akr	GE651674.1	23.8	[22]							
csi-miR414 h	GW863581.1	86.83	[22]							
miR156	HS396956.1	45.8	[23]	113	125	AAC	3	CGTTAGGCTATTTTGTTTCAA	TTCTGTCAATCATCCAATTTC	159
miR171a	FS948108.1	39.2	[23]	115	127	TC	2	TACTTCCAACCAAACACAAGT	TAGCTTACCACCTCAATCAAA	168
miR171b	FS948109.1	40.4	[23]							
miR397	CV699725.1	39.2	[23]							
miR399	FS958856.1	52.8	[23]							
miR2863	FS950435.1	21.5	[23]	57	69	TCTA	4	CTCCTGTACACTCTCTCTCTCC	GATGAACAGCATAGGTATCCA	127
miR2911a	JK476023.1	65.4	[23]							
miR2911b	FS953337.1	69.2	[23]							
miR5021a	GW690847.1	71.9	[23]	72	84	GGA	3	AGACACAGGCAGACATAGAGA	AAGATGCGATGAGATCAGATA	165
				239	254	TTC	3	AGATACACATTGGGAAGAAGG	TAGAACTTGCAGAGAGAAACG	149
				252	264	TC	2			
miR5021b	GE651759.1	72.2	[23]	60	78	GA	2	TAGAACTTGCAGAGAGAATCG	TACACATTGGGAAGAAGAAGA	152
				75	90	AGA	3			
miR5368a	GE653011.1	76.1	[23]							
miR5368b	FS945766.1	68.5	[23]							
miR6483a	HS398296.1	29.8	[23]							
miR6483b	JK714410.1	30	[23]							
miR1533	JK478587.1	89.99	Reported	403	419	AC	2			
miR8002-3p	FS955851.1	155.67	Reported							

Plant microRNAs do regulate the transcripts expression for growth, development and stress responses by altering leaf morphology and polarity, organ development, lateral root formation, hormone signalling, cell death, signal transduction, cell differentiation and proliferation, transition from juvenile to adult vegetative phase, vegetative to flowering phase, flowering time, floral organ identity and reproduction [41, 43–46]. Meanwhile, miRNAs are involved in the regulation of gene expression through mRNA cleavage or translational inhibition [47] has been reported, therefore obtaining information about target genes of potential microRNAs was an essential part of the study. Since the full genomic information is lacking in case of tea, DFCI Gene Index of *Arabidopsis thaliana*, *Glycine max*, and *Zea mays*, *Solanum tuberosum* etc. were used as target database. Among the presently reported two microRNAs, miR1533 were not found to have any target similarity with significant threshold values of input parameter. The other one, miR8002-3p were predicted to have cleavage activity on General transcription factor 2-related zinc finger protein (Target Accession: AT1G42710.1), phosphoglycerate mutase (Target Accession: TC194811). In addition the target genes of other predicted miRNAs of tea were explored from the literature to elucidate their role in pre-miRNA SSR based polymorphism assessment. Zhu and Luo [23] reported that their predicted miRNA target genes encoded transcriptional factors, involved in stress response, transmembrane transport, and signal transduction and transcription regulation. Target genes encoding transcription factors and cell integrity maintenance machinery during stress response was also mentioned by Prabu and Mandal [21]. Multiple target of a single miRNA is the system biology network, when they control expression of different transcription factors which in turn regulates specific genes for different metabolism [48].

Microsatellite markers are widely used tool for estimating the genetic variation and especially used for construction linkage map, understanding of marker trait association, identification of disease resistant loci [14, 49]. The distribution of simple sequence repeats or SSRs in the genome is inherently unstable and therefore highly polymorphic [50]. People assumed that increment of the repeat unit and repeat tracts gives rise the chances of the mutation rate [51]. Some reports have already established the fact that SSR expansions or contractions within genome sequences can affect functions of these sequences and even lead to phenotypic changes [17]. Evidences have shown the effect of SSR unit variation within protein coding regions. However, the consequences of the same in non-coding transcripts are less studied. A very few reports demonstrated the significance of analysis of SSRs in non-coding miRNA [12–15, 49]. In current study, 13 of 33 total predicted pre-miRNAs had one or more SSR motifs (Table 1). A total of 9 sequences had SSR motif in a single region, whereas 3 sequences contained SSR motifs in two locations and 1 sequence was with 3 different SSR motifs. Trinucleotide motifs were most abundant among all followed by dinucleotides. There was one each of penta and hexa-nucleotide motifs. Forward and reverse primers from the each microsatellite motifs flanking region could be generated in all members excluding only one where SSR motif present toward the terminal end. The microsatellite motifs may be conserved among group or become signature [13] which might be used in studies of genetic fingerprinting work of tea. Some traits rely on specific repeats of microsatellites and their numbers. Such advantage has been efficiently employed by rice researchers when miRNA-SSR markers were employed to differentiate the salt tolerant and susceptible genotypes [15]. They found more repeat variation of the salt responsive miRNA genes among the susceptible rice genotypes than tolerant one. Such extensive work can be applied in tea to distinguish the cultivars with varying agronomic traits on the basis of miRNA-SSR polymorphism.

Finally, newly predicted microRNAs in tea would enrich the assemblage in absence of whole genomic information of tea and make easier subsequent studies for experimental validation. Some of them might be related to certain metabolic functions thereby phenotypes as well. Excavating of microsatellite motifs from predicted microRNA precursors and designing primers from SSR flanking regions would pave the way in developing novel markers for tagging tea specific agronomic traits as well as accelerating non-conventional breeding program. This can freely be followed by genetic diversity assessment of tea cultivars with varying characters.
